# Preparation of the pre-service teacher to deliver comprehensive sexuality education: teaching content and evaluation of provision

**DOI:** 10.1186/s12889-024-18982-0

**Published:** 2024-06-06

**Authors:** Jacqueline Hendriks, Lorel Mayberry, Sharyn Burns

**Affiliations:** 1 Curtin School of Population Health, Discipline of Health Promotion and Sexology, Curtin University, Bentley, WA Australia; 2 Collaboration, for Evidence, Research and Impact in Public Health, Curtin University, Bentley, WA Australia

**Keywords:** Pre-service teacher, Comprehensive sexuality education, Initial teacher training, Australia

## Abstract

**Background:**

Despite the extensive benefits associated with the provision of comprehensive sexuality education (CSE) within a school context, many initial teacher training programs inadequately prepare pre-service teachers to deliver this content. Programs that do provide such instruction do not routinely share details of their curriculum, syllabi, or evaluation data.

**Methods:**

This paper outlines the structure of an Australian undergraduate course for pre-service teachers that focuses on instruction in CSE. This course spans twelve teaching weeks, aligns with evidence-based principles for sexuality education, prioritises experiential learning and requires students to complete authentic, practical assessment tasks. Formative, process, and short-term impact evaluation data, based upon five years of delivery, are described.

**Results:**

Students completing this course reported statistically significant improvements in attitudes associated with CSE and comfort in facilitating all domains of learning (knowledge, attitudes, skills).

**Conclusions:**

Positive process and short-term impact data provide strong evidence for the provision of CSE to pre-service teachers, regardless of future teaching speciality. Proposed amendments include the creation of a fully online tuition pattern and an expansion of content to incorporate other audiences, such as community-based educators.

## Background

Comprehensive sexuality education (CSE) is a clearly defined intervention that can provide children and young people with the knowledge, attitudes, and skills necessary to optimise their sexual health and wellbeing [1]. Internationally, the multiple protective benefits offered by CSE provision have been well established. When evidence-based CSE is provided, learners receive accurate and age-appropriate information about relationships, the human body, gender, sexuality, sexual activity, and sexual and reproductive health [1–4]. They cultivate critical skills related to communication, bodily autonomy and consent, decision making, media literacy, and help seeking for themselves and others [1–4]. Furthermore, CSE plays a pivotal role in affirming diversity and challenging harmful stereotypes and norms [1–4]. This can help to foster inclusivity and combat discrimination towards a range of factors such as sex, gender, sexuality, religion, culture, and disability [1].

Repeated international evidence reviews have declared schools to be critical sites for the provision of comprehensive sexuality education (CSE) to young people in an age-appropriate way across all year levels [1–4]. School-based CSE, as a complement to home-based or community-based learning opportunities, is highly regarded because most young people spend a significant proportion of their time at school and view this setting as a trustworthy source of information. Furthermore, schools provide a cost-effective opportunity for young people to receive learning materials that are developmentally appropriate, consistent with their peers, cognisant of their personal or family circumstances, and can link them (or their families) with local services [1–4].

Unfortunately, the inadequate and ad hoc preparation of teaching staff to deliver CSE to students is a barrier to comprehensive delivery [5]. While some examples of initial teacher training in CSE have been published [6–8], teacher training organisations do not routinely share this information [9], and teacher preparation is known to vary widely [10].

Reviews that consider the student perspective have highlighted issues with classroom teachers as the providers of CSE [2, 5]. This may be overcome with appropriate training at the pre-service level, alongside ongoing support, and professional development at the in-service level [5, 11, 12]. Efforts to strengthen initial teacher training programs, to ensure sexuality-related content is provided to all pre-service educators, may be bolstered in jurisdictions that mandate the provision of such content to their students [13, 14]. Finally, several groups have championed for minimum teaching preparation standards in regard to the provision of sexuality education [15–20], and some exemplars have been published [21, 22].

Within the Australian context, although the national curriculum and more than 20 national strategies, plans and senate reports, either supports or directs the provision of CSE, there has been no effort to standardise or scale up teacher training in this area [23]. Presently, initial teacher training programs do not routinely provide instruction on CSE [9, 24]. This results in differing levels of teaching confidence and competence, leading to wide variability in how CSE is provided across the country, and within individual schools [25]. Historically, there have been requests for all pre-service teachers, regardless of their teaching speciality, to receive instruction in human sexuality [24] and calls for the establishment of teacher preparation standards [20].

Since 2014, the *Curtin University RSE Project* [Relationships and Sexuality Education Project] has received funding from the Western Australian Department of Health to provide professional development services in CSE to pre-service and in-service teachers throughout the state. The phrase ‘relationships and sexuality education’ was specifically chosen, as this is the nomenclature used in Australian school curricula to encapsulate CSE issues. The *RSE Project* is a partnership between the Collaboration for Evidence, Research, and Impact in Public Health; the School of Population Health; and the School of Education at Curtin University, Australia. Some of our work with in-service teachers has been published elsewhere [11, 26].

To provide professional development support to pre-service teachers, an undergraduate elective course^1^ focused on the delivery of CSE was developed. The purpose of this paper is to present key data related to this process. The manuscript is structured in three phases: (i) formative evaluation (to describe the development process); (ii) initial teacher training course in comprehensive sexuality education (to provide a summary of the course content and our methods of teaching and learning instruction), and (iii) process and short-term impact evaluation (to provide quantitative and qualitative data spanning five years of delivery). For phase three, the research question sought to explore the impact of the preservice teacher training course on participant confidence and perceived skills to implement CSE.

### Course development

Formative evaluation was undertaken to inform the development of the course learning outcomes, teaching and learning content and assessment tasks. This included focus group discussions (FGDs) with undergraduate education students (described below). Furthermore, key materials were informed by a review of the academic and grey literature, a review of current undergraduate and postgraduate courses in sexology offered by Curtin University, and the personal expertise of the authors. All named authors have extensive expertise in CSE, including teaching and learning, developing curricula and providing professional development services. In particular, the course coordinator LM drew upon their extensive experience as a primary school, tertiary, and community-based sexuality educator to finalise the course content.

In alignment with contemporary evidence-based guidelines for the provision of CSE, the course prioritises participatory, collaborative, and learner-centred teaching and learning methods that assist young people to internalise and integrate information [1, 5, 12]. Intercultural, gender transformative and human rights perspectives are also highlighted throughout, alongside a positive approach to human sexuality [1, 5, 12].

The course was reviewed by the Directors of Teaching and Learning for the Schools of Population Health and Education, as well as the Curtin University Academic Board before it was first offered to students. It is also subject to ongoing review as part of regularly scheduled university-based reviews.

## Materials and methods

Undergraduate students enrolled in an education degree at any Western Australian university were recruited via opportunistic and snowball sampling to participate in a series of FGDs. The semi structured interview schedule explored the level of intention pre-service students had towards delivering CSE in their future classrooms, their level of confidence to do so, the specific topics they felt should be covered in an undergraduate CSE course, and the types of assessment tasks they perceived to be most relevant for them.

FGDs were conducted in public meeting spaces on various university campuses and were audio-recorded and transcribed verbatim. Light editing was used to ensure readability. Thematic analysis was conducted to highlight recurring themes [27], and a formalised coding process was followed [28]. Preliminary coding was conducted by LM and verified by all authors. As the FGDs were structured to identify specific issues related to course development, a deductive approach was employed.

## Results

Fifteen undergraduate students (four male, ten female, and one transgender) participated in the FGDs. Most were aged 18–26 years; however, three were older. Participants represented all four universities in Western Australia that were providing tertiary training in education and were completing degrees in early childhood (*n* = 2), primary (*n* = 6) or secondary (*n* = 7) education. FGDs lasted approximately 45–60 min. Three themes were derived from the data to help understand intention and confidence to deliver CSE, as well as specific content and assessment tasks to incorporate in the undergraduate course.

### Intention and confidence to deliver CSE

Eight participants did not feel confident discussing sexuality with their future students, and some cited that their current undergraduate program had not yet covered this topic:

“We did a health and physical education course, and it was useless. Almost nothing about health at all and nothing that was at all controversial like sex or drugs” (primary pre-service teacher);

“We haven’t learned anything about the curriculum or where to get lesson ideas from. Is there any materials that make this a little easier?” (primary pre-service teacher);

“I would freeze. I don’t think all teachers are up to teaching about sex. This hasn’t been covered in our degree. I think students go and see the school nurse if they have those sort of problems and questions” (secondary pre-service teacher);

“I have no idea about what to say and where to start” (secondary pre-service teacher);

“I wouldn’t know what to teach or how to approach it. I haven’t seen it taught when I have been on practicum” (secondary pre-service teacher); and.

“I think this should be covered at university in our teaching degree. We have covered nothing about sexuality. At this point, I think it is something I would avoid if I could” (secondary pre-service teacher).

Some participants were adamant that discussions about sexuality would not be necessary in their future roles because they would be teaching younger children, specialising in particular roles or working in religious schools:

“I don’t think sexuality is part of a pre-schooler’s life. I don’t know, but I think that sort of topic starts at high school” (early childhood pre-service teacher);

“Young children don’t need to know about sex, so it’s not really relevant for me” (primary pre-service teacher);

“I don’t feel competent at all. I will be working in a Catholic School, and they don’t teach about sex” (secondary pre-service teacher);

“I don’t think all secondary school teachers have to teach this subject. I think the physical education teachers do that. And that’s not me” (secondary pre-service teacher); and.

“I will teach maths and science subjects. I don’t want to teach that” (secondary pre-service teacher).

Some participants indicated that they were fearful of parent reactions:

“I have no sexuality education from my parents. I also don’t think parents of students are in favour of their children knowing too much about sex too early” (primary pre-service teacher); and.

“I don’t think the parents would like that being discussed. No, I don’t feel confident” (secondary pre-service teacher).

Overall, nearly half of the participants stated or implied that they did not plan to provide CSE in their future classrooms. Reasons included perception that CSE concepts should not be taught in early childhood or primary year levels, a general feeling of discomfort with the subject area, a belief that students would be “*difficult to control*” in CSE classes or that questions would be embarrassing, fear of the reaction of others (e.g., parents, other teachers), a lack of CSE role models, and a lack of knowledge about CSE content and strategies.

Seven participants indicated some degree of confidence in discussing sexuality; of these, five had already completed at least one of the two undergraduate sexology courses already offered by Curtin University:

“I have studied sexology, and I feel entirely comfortable, but I don’t think I am the norm. I think many adults think children and teenagers aren’t ‘ready’ to talk about sexuality or that it isn’t appropriate, or that they’ll offend someone and it’s all too hard. Not talking about sex with young people doesn’t make them not do it, it just makes them do it unsafely, or feel shame because they think they’re abnormal, or hide something because they’re afraid to ask or misunderstand something about their own development. I believe all aspects of sexuality should be covered in schools. It is an integral part of being human. It is the job of the adults, including the parents, to make it safe and comfortable to talk about” (secondary pre-service teacher).

It should be noted that these elective courses are relatively unique and not commonly offered by universities worldwide. While they address foundational and contemporary concepts in sexology, their focus is not on the delivery of CSE within school settings.

Another student indicated that they felt confident discussing sexuality with their future students because “*mum was a nurse and taught me about my body”* (primary pre-service teacher).

### Specific topics to be covered in an undergraduate CSE course

When asked what topics should be included in a CSE course, many participants focused on the biological aspects of sexuality, suggesting topics such as anatomy, puberty, reproduction, contraception, and sexually transmissible infections. However, other suggested topics included *“the internet, media, sexting and pornography,” “emotional topics such as rejection, first-time sex or staying a virgin,” “organisations that can help teachers,”* and *“gender roles and diversity.”* These suggestions were provided by multiple participants enrolled in courses from early childhood through secondary education.

Two participants who had completed a sexology course previously stated:

“I think this education would make a big difference when it comes to children and sexual abuse. Date rape and other risky events should be discussed. Plus, personal hygiene, awareness of sexual infections, contraception and simply knowing more about your own body. Talk about pleasure, not just the negatives. Knowing that everyone is different and deserves respect. We are all sexual beings” (primary pre-service teacher); and.

“I would like to see the elimination of the words ‘dirty’ and ‘wrong’ within an adolescent’s thoughts towards sexuality. They need to learn to feel comfortable with their thoughts and feelings about their own sexuality. I would also like to learn how to teach about things like pleasure and intimacy as well as the basic sexually transmissible infection and pregnancy information that students are given” (secondary pre-service teacher).

These two respondents demonstrated a broader awareness of sexuality beyond the biological aspects. They highlight the benefit of taking a positive approach to sexuality education with opportunities for examining attitudes and values related to sexuality, affirming diversity, and reducing risky behaviours.

While most participants focused on specific sexuality topics, some requested information on relevant resources, strategies, and curricula. Almost half of the participants wanted the course to provide information on how to create a safe or conducive learning environment to discuss CSE topics. One student requested skills to formally assess CSE teaching and learning.

### Relevant assessment tasks

Participants provided a broad range of suggestions in relation to possible assessment tasks. However, most were adamant that the tasks needed to be “*practical,” “real”* and *“something we can actually use in our practicum placements or in our future classes.”*

Opinions towards oral presentations were mixed. While some suggested individual or group presentations to showcase a large range of ideas, some participants clearly cited their dislike for group work, such as this response:

*“Anything but group projects. They are unfair for the hard workers, and others can cruise”* (early childhood pre-service teacher).

Participants recommended the collation of teaching and learning resources such as pamphlets, websites, worksheets, and lesson plan ideas. Similarly, many participants felt that writing term programs or lesson plans would be practical assessment tasks that they could readily take to future classrooms. Research papers on specific CSE topics were suggested by five participants and an online test by three participants.

### Initial teacher training course in comprehensive sexuality education

### Course learning outcomes

Since 2014, this elective undergraduate course in CSE has been available for any student studying an Education course at Curtin University. It is also available to students within the Faculty of Humanities who may select it as an elective. On successful completion of this course, the course learning outcomes indicate that a student will be able to:

1. plan, and present creative, interactive and inclusive relationships and sexuality education lessons using appropriate curriculum;
2. apply relationships and sexuality education research for the development of school education programs;
3. critically reflect and discuss aspects of sexuality in a comfortable, respectful and nonjudgmental manner; and.
4. analyse and apply methods for teaching relationships and sexuality education.

### Key topics and mode of delivery

The course is delivered over twelve teaching weeks, with three hours of interactive classroom instruction each week. A series of required readings are drawn from the contemporary literature, book chapters and peer-reviewed journal articles focused on evidence-based provision of CSE.

The current course coordinator and lecturer (author LM) has undergraduate and postgraduate qualifications in Education and a PhD in Sexology. In addition to delivering this course since its inception, they have over 40 years of experience in delivering education and sexology content to tertiary students, in addition to educating in-service teachers, professional groups and the general community. The course covers a broad range of topics, summarised in Table 1.

**Table 1:** Content of the undergraduate comprehensive sexuality education course for pre-service teachers

Weekly content	
• background to CSE including an explanation of key terms, current evidence-based practice and key statistics • self-reflection activities to consider ‘you’ as a CSE educator • important guiding documents (e.g., government policies, curriculum, whole-school delivery) • current discourse in school-based CSE and the importance of a sex-positive approach • creating a safe learning environment for school-based CSE • interactive teaching and learning strategies for all year levels from kindergarten to year 12 • affirming diversity • respectful relationships, consent and enhancing friendships • gender, power and control • prevention of child abuse or neglect/protective behaviours • anatomy, puberty, menstruation, reproduction • pleasure • safer sex, sexually transmissible infections • social media, sexting and pornography • teaching CSE in a variety of cultural settings • community-based organisations and other resources that can further support CSE delivery • evaluation and assessment of CSE	

All course content is explicitly mapped to current Australian professional standards for teachers, which is a document outlining seven overall graduate standards and 37 specific foci, to ensure high-quality teaching and learning [29]. Throughout this course, all standards and most foci are addressed to varying degrees.

### Assessment tasks

Students are required to complete three assessment tasks and obtain an overall score of 50% or higher to pass the course. Task one is to complete a non-invigilated online test (worth 20%) within a 60-minute timeframe. This test comprises multiple choice, multiple answer and short answer questions that are designed to assess a student’s awareness and comprehension of the required readings and ability to locate relevant CSE resources.

The second task is a resource file (worth 50%) comprised of three sections. Each week, students summarise the various energisers, grouping strategies, teaching and learning strategies, resources and community-based organisations that are demonstrated or discussed during class. Next, they select and critically review an Australian website that would be relevant to their future students or to their delivery of CSE. Finally, they write a two-page reflection paper outlining how they responded to the weekly content, their viewpoints on CSE and how this may have changed throughout the semester, and their overall experience of the course.

The final assessment task is a group presentation, typically completed in triads, delivered to the rest of the student cohort during the final two weeks of the semester (worth 30%). Students are required to plan and deliver a 25-minute CSE lesson designed for a specific age group (from kindergarten through to year 12). Issues such as literacy, disability, cultural sensitivities, and age are identified and considered in the planning. There is a focus on planning and delivering a lesson that is interactive and engaging. Students are expected to contribute equally to this assessment task, and each group member individually submits a review of their peers. Typically, each student in a group receives the same grade for this assessment task, unless the peer review highlights that the workload was not shared equally. The final lesson plans are also shared with the entire student cohort to enable them to build their teaching portfolio.

### Teaching and learning strategies

In addition to the required readings, students are expected to attend the weekly 3-hour face-to-face seminar. The ideal room layout is a flat floor classroom with enough space for interactive activities and a maximum class size of 30 students. The seminar can be livestreamed and recorded so that students who are unable to attend the session in person can access it; however, face-to-face attendance is strongly encouraged.

Like prescribed course content, teaching and learning strategies are specifically engaged to address graduate teaching standards [29]. Various teaching and learning paradigms, such as critical pedagogy (e.g. 30, 31), social constructivism [32, 33], and experiential learning (e.g. 34), underpin all classroom activities.

To model a safe learning environment [1], a group agreement is made collectively during the first class and routinely referred to throughout the semester. Typically, this agreement encourages students to be open to the opinions of others, to use “I” statements rather than speak on behalf of others (e.g., “I feel this lesson would be difficult for me to teach because…” as opposed to “teachers would find this lesson difficult to teach because…”), to respect confidentiality and to engage respectfully in class.

Critically, all group agreements give students the right to pass should they not wish to participate in an activity or respond to questions. The lecturer will routinely ask open-ended questions to the class, working hard to elicit responses from all students, but will not actively target any one individual to respond or share their opinion.

As per best-practice guidelines for quality CSE provision [1], the course focuses on showcasing how CSE can be delivered in interactive and engaging ways, as opposed to didactic instruction. All domains of CSE learning, including knowledge, attitudes and skills, are addressed. Every week, the lecturer will continually group and regroup students using various strategies, showcase interactive teaching and learning strategies applicable for a variety of ages, learning abilities, and cultural contexts, and highlight numerous resources. Over the course of a semester, students become experiential learners, participating in a range of strategies that include ice breakers, roleplay, dilemmas, group brainstorms, model making, stimulus cards and the use of multimedia (e.g., popular music, cartoons, television shows, fiction and nonfiction texts, advertisements, movie clips, online videos). Cooperative learning strategies such as paired sharing, triads and group work are modelled. Once activities have been modelled, the lecturer then provides open-ended processing questions so that students can consider [1] when such an activity or strategy may or may not be appropriate [2], how the activity could be adapted, and [3] whether there are any issues with delivery that require specific attention.

Throughout the semester, concepts of self-awareness and self-care are paramount. Students are asked to consider their own personal backgrounds and learning, as they may relate to CSE, and to consider how these perspectives may or may not impact their ability to facilitate CSE content. This process is similar, but not equal to, a Sexual Attitude Reassessment and Restructuring (SAR) training program [35]. These more formalised programmes engage participants in a process of exposure and self-reflection, so they may consider how attitudes and values towards sexuality may influence their professional practice. Current SAR guidelines require 10–16 h of instruction [35]; hence, it was not possible for this course to provide this level of instruction. However, other undergraduate and postgraduate sexology courses currently offered at Curtin University are equivalent to a comprehensive SAR program. Some pre-service teachers, who had elective available in their degrees, may have enrolled in such a course.

Techniques to manage self-care are also identified in the first class and are routinely addressed throughout the semester. Self-care was prioritised, as CSE content has the potential to elicit strong emotional responses [36] and is an important consideration in trauma-informed practice [37]. Students create their own self-care plans and identify strategies to support their body, mind, and spirit throughout the semester. The ‘right to pass’ and adherence to other group agreement principles is also seen as an important self-care strategy. Students are supported in taking a break if any of the content raised during the semester is triggering or upsetting. To further support this process, the lecturer provides trigger warnings prior to the delivery of any material with the potential to cause distress (e.g., lessons on child sexual assault or domestic violence). Access to free university counselling services are also promoted. Finally, pre-service teachers are encouraged to apply these self-care skills beyond the present context, to maintain them as in-service teachers and facilitators of CSE and to educate their future students about the importance of self-care.

### Process and short-term impact evaluation

## Materials and methods

Students enrolled in the undergraduate CSE course offered by Curtin were invited to provide various forms of feedback during their participation in the course. First, all students who complete a tertiary course at Curtin University are invited to complete an online survey, known as *eVALUate*, to provide feedback regarding content, delivery, and assessments. As the questions in this survey are predetermined by the university and are generic for all courses, we supplemented this with an additional online cross-sectional survey administered prior to the first class and immediately post-course. These survey items sought to capture pre-service teachers’ attitudes towards CSE, to self-rate their knowledge of and comfort with the subject area and their ability to facilitate CSE lessons and to provide specific feedback regarding the course content and assessment tasks. For some items, open-ended text responses were requested. All *eVALUate* and survey data were anonymous.

Data over a five-year period were collated for analysis. For quantitative data, descriptive statistics are provided. Unpaired t-tests were performed on mean scores collected pre- and post-course. Mean, standard deviations, significance and 95% CI are shared. For qualitative data, thematic analysis was conducted [27, 28]. Preliminary coding was conducted by LM and verified by all authors.

## Results

Over five years of delivery, 176 pre-service teachers completed the CSE course. All students received a pass mark or higher. A summary of key demographic information is provided in Table 2. The majority of students were female (87.5%) and enrolled in Bachelor of Education (Primary) (55.1%). Fifteen students were enrolled in a course that would not result in a direct teaching qualification. These were a Bachelor of Arts (*n* = 11), a Bachelor of Psychology (*n* = 2), a Bachelor of Educational Studies or a Bachelor of Science.

**Table 2:** Key characteristics, all enrolled students

		*n* (%)
Gender	Male	22 (12.5)
	Female	154 (87.5)
Enrolled course	Bachelor of Education (Early Childhood)	51 (28.9)
	Bachelor of Education (Primary)	97 (55.1)
	Bachelor of Education (Secondary)	13 (7.4)
	Bachelor of Arts	11 (6.3)
	Other	4 (2.3)

### Evaluate data

**Fig. 1:**
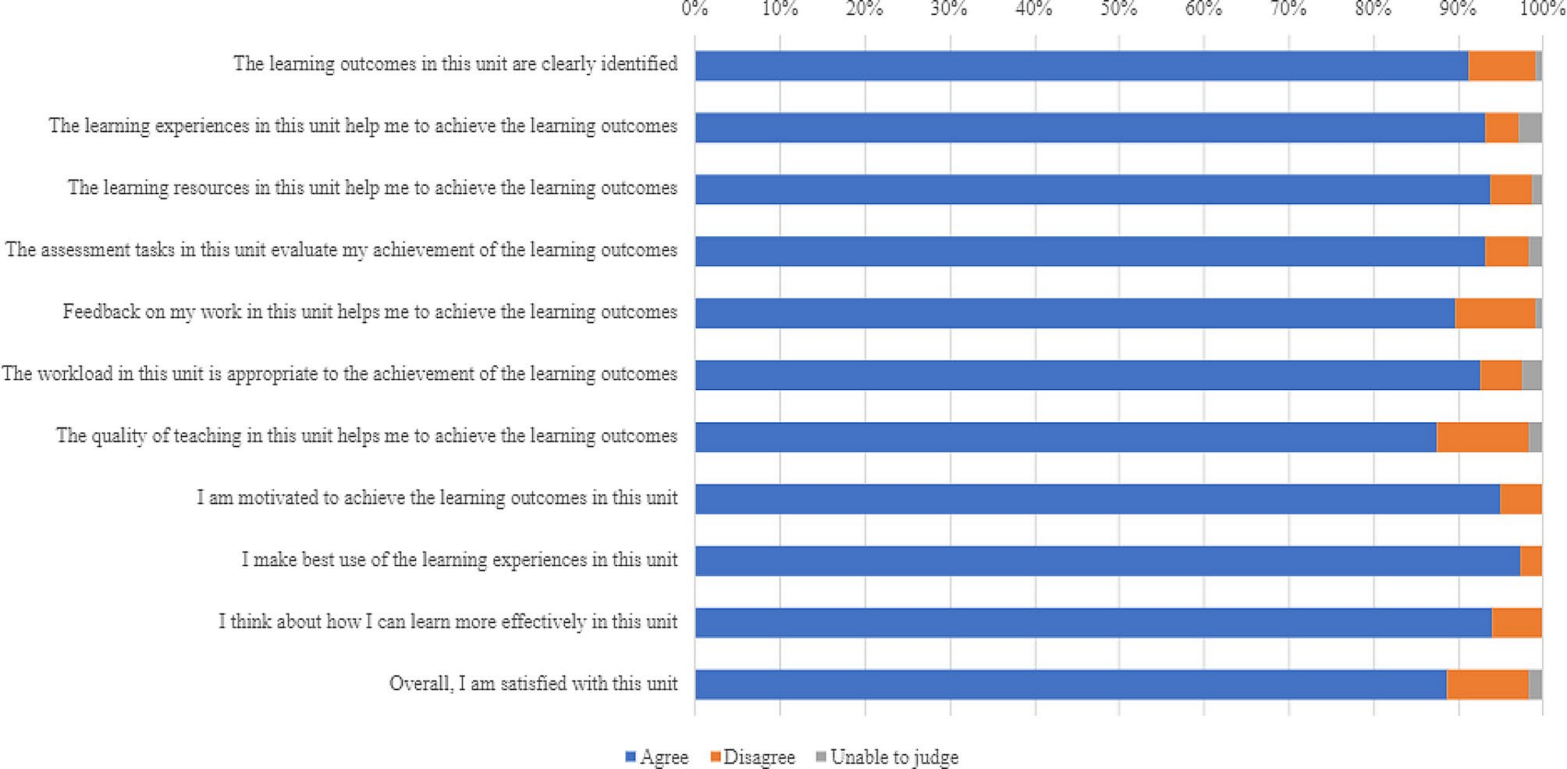
Evaluate data [Higher resolution image provided separately]

Collated eVALUate data were based on an overall response rate of 37.5% (*n* = 66 students). As illustrated in Fig. 1, there was strong agreement or support for all items (87.4% or higher).

### Student survey data

#### Quantitative data

Survey data from students provided an overall response rate of 88.0% (*n* = 155 students) at baseline and 43.75% (*n* = 77 students) post-course. A further demographic breakdown is provided in Table 3.

**Table 3:** Key characteristics, survey respondent sample

		Baseline	Post-course
Total		155 (100)	76 (100)
Gender	Male	13 (8.4)	5 (6.5)
	Female	140 (90.3)	71 (93.5)
	Other	2 (1.3)	0 (0)
Age	17–21 years	94 (60.6)	44 (58.4)
	22–26 years	48 (31.0)	25 (32.5)
	27–31 years	9 (5.8)	5 (6.5)
	32 + years	4 (2.6)	2 (2.6)
Enrolled course	Bachelor of Education (Early Childhood)	45 (29.0)	22 (29.9)
	Bachelor of Education (Primary)	81 (52.3)	43 (55.8)
	Bachelor of Education (Secondary)	12 (7.7)	5 (6.5)
	Bachelor of Arts	14 (9.0)	6 (7.8)
	Other	3 (1.9)	0 (0)

At baseline, 9 students indicated that they had participated in some level of CSE training prior to enrolment in this course. This included enrolment in a previous sexology course at Curtin University or training from a local nongovernment organisation (e.g., WAAC, Youth Affairs Council of Western Australia, Curtin University RSE Project).

Based on five attitudinal statements, students demonstrated a positive change in attitudes between baseline and post-course, based on a change in mean values (*R* = 1–5). All changes were statistically significant (refer to Table 4).

**Table 4:** Attitudes related to school-based relationships and sexuality education

	Mean baseline value (SD)	Mean post-course value (SD)	Sig (95% CI)
Children learn about sexuality from the time they are born	3.41 (1.10)	4.21 (0.94)	*p* < 0.0001(-1.09, -0.51)
I understand that my own values, beliefs and attitudes around sexuality affect my work with young people and my peers	4.05 (0.89)	4.45 (0.79)	*p* = 0.011(-0.63, -0.16)
Using health-trained teachers, working in partnership with parents and the wider community, is the ideal way to provide good practice relationships and sexuality education in schools	4.25 (0.73)	4.58 (0.74)	*p* = 0.0016(-0.53, -0.13)
The majority of parents support relationships and sexuality education in schools	2.92 (0.94)	3.64 (0.95)	*p* = 0.0001(-0.99, -0.47)
Relationships and sexuality education is a vital part of health education for all students K-10	4.2 (0.88)	4.63 (0.67)	*p* = 0.0002(-0.66, -0.21)

Students were asked to rank their abilities at baseline and post-course. There was a significant improvement in their knowledge of the subject area, comfort with the subject area, facilitation skills and desire to teach CSE (refer to Table 5). At the conclusion of the course, students were presented with the statement “*I feel that this course has improved my capacity to deliver relationships and sexuality education in a school setting*?” Amongst all respondents (*n* = 76), 73.6% strongly agreed and 14.5% agreed with the statement.

**Table 5:** Comfort to deliver school-based relationships and sexuality education

	Mean baseline value (SD)	Mean post-course value (SD)	Sig (95% CI)
I feel comfortable teaching students about specific information in a relationships and sexuality education context (e.g. intimacy, STIs, conception, contraception) as developmentally appropriate	3.37 (1.12)	4.21 (0.84)	*p* < 0.0001(-1.13, -0.56)
I feel comfortable facilitating discussions about values, beliefs and attitudes in a relationships and sexuality education context (e.g. feelings about lesbians and gay people) as developmentally appropriate	3.55 (1.12)	4.26 (0.88)	*p* < 0.0001(-1.00, -0.43)
I feel comfortable facilitating skills activities in a relationships and sexuality education context (e.g. decision making, communication, goal setting, stress management) as developmentally appropriate	3.56 (1.03)	4.37 (0.83)	*p* < 0.0001(-1.07, -0.54)
I am looking forward to facilitating a relationships and sexuality education class in a school setting	3.74 (1.03)	4.37 (0.67)	*p* < 0.0001(-0.89, 0.38)

#### Qualitative data

Finally, at baseline and post-course, students were given the opportunity to provide open-ended comments regarding their knowledge of the subject area, their comfort or confidence with the subject area, and their facilitation skills.

At baseline, most students expressed limited knowledge regarding CSE. This was often due to a lack of education from home or at school. Some example statements include:

“My knowledge around sexuality and relationships is very poor because I have not had any training or exposure to classes around this topic, since health lessons in school (which was a number of years ago)” (Bachelor of Education, Early Childhood Education); and.

“I have minimal knowledge of the subject area. This is a subject that was rarely touched on in my school years and by my family” (Bachelor of Education, Primary Education).

However, some students at baseline expressed that their knowledge levels were “adequate” or “reasonable.” Other students indicated that while they knew about many CSE topics, their knowledge was mostly gained from school and personal experiences, and they were unsure how to necessarily apply this to a school context:

“I am aware about the subject area, however, feel like I need a recap and a refresh on some of the topics to ensure my knowledge is correct and current before I feel confident enough to facilitate a class on this subject” (Bachelor of Education, Early Childhood Education);

“My knowledge of sexuality and relationships education is probably intermediate as I have learned it through schooling and experience and witnessing of the world and am familiar with most aspects of sexuality and relationships” (Bachelor of Education, Primary Education);

“I’m eager to learn about sexuality education, I think that it is very important, but I know that I don’t know a lot of information or skills that would be developmentally appropriate, or how to approach CSE in a classroom” (Bachelor of Education, Primary Education);

“I have an average knowledge about sexuality and relationship education. I’ve been taught in high school. I feel comfortable and confident with this subject area” (Bachelor of Education, Secondary Education);

“I think I may be competent enough to teach sexuality and relationships education to pass on general knowledge but am not confident enough to address students concern or accurately answer every question” (Bachelor of Education, Secondary Education); and.

“I consider myself to have a comfortable level of knowledge through studying courses in sexology and personal research; however, I am aware that I need and would like a lot more knowledge before undergoing an official CSE teaching role” (Bachelor of Arts, Humanities).

Overall comfort to address sexuality attitudes and to facilitate CSE was very low. For most, this was due to a lack of role modelling or practical experience.

“I wouldn’t feel very confident teaching to children at the moment, but hopefully this perspective changes after this course” (Bachelor of Education, Early Childhood Education);

“If I had to teach a class this subject tomorrow, I would have no clue where to start!” (Bachelor of Education, Primary Education); and.

“My confidence in teaching the subject is very low as I would be nervous in my approach to the topics and worried people may disagree with my teaching or parents may be angry at me for teaching it in general” (Bachelor of Education, Primary Education).

Open-ended comments provided post-course highlighted an increase in confidence to deliver CSE for all students, with participants suggesting they looked forward to facilitating engaging and interactive lessons. Some also expressed that the course provided them with valuable skills to discern appropriate resources and to support other teachers.

“I have learnt an exceptional amount of information throughout this course and because of the facilitator who created a very comfortable learning environment. I now feel very comfortable in facilitating and educating others on the various topics covered within relationships and sexuality education” (Bachelor of Education, Early Childhood Education);

“After completing the course, I feel very confident in my own knowledge base about the topic and inspired to bring that knowledge into the classroom. After taking the course, I also hope to find myself in a health role within a school. The course provided us as teachers with vital information to teach children. I feel confident in my facilitation skills teaching these topics in a fun and engaging way that will work to reshape the way people think about relationships and sexuality education. I feel like I have learnt a lot about the subject area that will stick with me because it was taught in an engaging way, and it was interesting. Thank you for the opportunity to take the course. I strongly recommend it to any teacher either training or in practice” (Bachelor of Education, Primary Education);

“I feel that I have gained not only knowledge of how and what to teach for CSE but also where I can get further information for myself as a teacher and others. I feel that due to this increase in knowledge, I have a greater understanding of my responsibility toward the students I teach. This in turn has provided me with the confidence to communicate openly and honestly about CSE subjects at a level that is appropriate for the students I am in contact with” (Bachelor of Education, Primary Education);

“I feel that I still need experience within the classroom to expand on my facilitation skills, as I believe that this skill MUST be learned through experience - there is only so much you may learn in the classroom and as a 3rd year university student, I still have 20 odd years to catch up to the facilitation skills shown by those teaching” (Bachelor of Education, Primary Education); and.

“I was taught how to teach these often uncomfortable topics and fun and engaging ways. Students will be excited to be involved rather than wishing they were not there” (Bachelor of Education, Secondary Education).

## Discussion

International reviews of initial teacher training programs have found limited examples of focused instruction in CSE, and of the training that was identified, information regarding teaching and learning materials was not readily available [9, 10]. Beyond focused instruction, it is widely documented that initial teacher training programs provide no or very limited support for pre-service teachers to provide any form of CSE to their future students [15, 18, 38–41]. To build workforce capacity, this paper summarised the teaching and learning content included within an initial teacher training course focused exclusively on CSE. The formative evaluation activities that informed the development of this course were outlined, and various process and short-term impact data were also shared.

The course outlined in this publication, supported by the reported evaluation data, indicates close alignment with evidence-based guidelines for the delivery of CSE [1, 42], Australian professional standards for teachers [29], and various teaching and learning paradigms (e.g. [30–34]). Teaching and learning methods focused on participatory, collaborative and learner-centred methods, emphasising human rights perspectives [1, 42].

Overall, there was consistent evidence that this course greatly improved the knowledge, attitudes, and skills of pre-service teachers to deliver CSE. These positive results closely replicate the findings of similar studies. Other educators who received formal training in CSE have reported improvements in knowledge about sexuality-related issues; and greater confidence or skills to create supportive learning environments, address sexuality-related issues in the classroom, facilitate broad and respectful discussions, and to affirm diversity [2, 6, 11, 18]. Furthermore, pre-service teachers expressed greater self-efficacy and intent to deliver CSE, which has also been reported by others who have also provided initial teacher training in this area [6, 18, 38, 43, 44].

Effective CSE requires delivery from educators who have the knowledge, confidence and pedagogical skills required to teach this unique content area [45]. It may also require educators to ensure that the core competencies they develop are cognisant of specific CSE issues [21]. For example, facilitators of CSE should value CSE as a human right; understand the inequitable and oppressive systems that impact CSE and sexual health; affirm a broad range of visible and invisible identities; be guided by an ethical framework that ensures decision-making is grounded in policy and prioritises health and well-being; plan a scope and sequence of activities that aligns with curricula, developmental stages, and the physical and cognitive abilities of students; effectively manage a range of student reactions; and collect assessment and evaluation data that are equitable, inclusive and culturally responsive [21].

To achieve this, a student-centred programme was developed that was highly practical and experiential in nature. Throughout the semester, there was a focus on affirming diversity, developing sex-positive attitudes, providing contemporary content knowledge, sharing best-practice guidelines, and demonstrating a variety of effective strategies for teaching, learning, evaluation, and assessment. All content was delivered by a highly qualified facilitator with expertise in education and sexology.

### Strengths, limitations and future directions

As previously noted, contemporary examples of initial teacher training programs that focus on CSE are difficult to access. The findings highlight positive short-term effectiveness of this course in enhancing confidence and skill development. It should be of great interest to these training programs to see such a program clearly articulated and supported with additional evaluation data. The data presented were limited to short-term metrics, and pre- and post- data were not matched. Future longitudinal studies, following educators throughout their journey from pre-service education into classrooms, is required to fully understand the effectiveness and efficacy of facilitating CSE in a school setting.

However, despite the ongoing success of this course, historically, degree structures within the Bachelor of Education (Early Childhood), Bachelor of Education (Primary) and Bachelor of Education (Secondary) at Curtin University provide limited opportunities for students to enrol in this course and this has impacted enrolment numbers. In particular, pre-service teachers who wished to work in secondary schools did not routinely select this course, as they had minimal electives within their degree structures. Therefore, the process and short-term impact data shared within this paper predominantly involved early childhood and primary specialists. Recent restructuring within the university has now ensured that this course is core for students enrolled in the newly created Bachelor of Education (Secondary) who are majoring in Health and Physical Education.

A fully online version of this course was first offered in 2023, and this is likely to increase enrolment numbers. The reconfiguration of this course, to suit an online audience, has been carefully curated by the teaching team and is based on significant experience in delivering highly interactive, student-centred content to health promotion and sexology students [46]. Course content is also being expanded to include the context of community-based education and to provide in-service education via postgraduate courses and a micro credential program (see Curtin Credentials: https://study.curtin.edu.au/professional-development/curtin-credentials/). These adaptations seek to ensure that a diverse range of educators and other pastoral care staff develop professional expertise to deliver evidence-based CSE in a range of school and non-school settings.

Data collected in this study may have been vulnerable to self-selection bias. Students elected to enrol in this course, and furthermore, they consented (or abstained) from providing feedback. It is therefore reasonable to assume that those with a specific interest in CSE elected to complete the course and those with stronger experiences (positive or negative) were more motivated to provide feedback.

Moving forward, the authors strongly advocate that all initial teacher training programs provide explicit instruction in CSE pedagogy to any trainee teacher likely to deliver CSE content to students in an early childhood, primary or secondary school context. Furthermore, regardless of future teaching specialties, *all* educators should be equipped with a contemporary understanding of the key CSE issues faced by young people. They should also develop skills to affirm diversity and ensure that the personal and social development of students is supported. Importantly, shame and stigma surrounding sex, sexuality and sexual health could be markedly reduced if young people can receive positive messaging and role modelling regarding CSE from a variety of adult sources within a school context, within a classroom context and throughout the school more generally. Finally, to further build the capacity of school systems to deliver evidence-based and comprehensive CSE, it is incumbent upon teacher training providers to share details of their programs more widely.

## Conclusion

In an effort to ensure that more initial training programs expand on their provision of CSE, this article has shared the formative process that underpinned the development of such a course within the Australian context. The various topics, teaching and learning methods, and assessment tasks that spanned twelve teaching weeks were clearly outlined. The positive process and short-term impact data that were collated provide strong evidence for the utility of such programs moving forward.

## Data Availability

The interview schedules, survey instruments and resultant datasets described in this manuscript are available from the corresponding author on reasonable request.

## Footnotes

1 Throughout Australia, an academic module typically spanning one semester of study is called a *unit*. However, most international institutions would refer to this as a *course* and so this is the term that has been utilised throughout this publication.
